# Impact of adverse events on survival outcomes in patients treated with CDK4/6 inhibitors for advanced breast cancer

**DOI:** 10.1007/s00280-025-04836-y

**Published:** 2025-12-03

**Authors:** Martina Catalano, Gestiana Cekrezi, Irene De Gennaro Aquino, Delia Ravizza, Alexandra Paulet, Kristian Shtembari, Claudia De Angelis, Roberto Petrioli, Daniele Generali, Giandomenico Roviello

**Affiliations:** 1 Department of Health Sciences, Clinical Pharmacology and Oncology Section, University of Florence, Florence, 50139 Italy; 2 School of Medicine and Surgery, University of Florence, Florence, 50134 Italy; 3 Clinical Oncology, Careggi University Hospital, Florence, Italy; 4 Medical Oncology Unit, Careggi University Hospital, Florence, Italy; 5 Unit of Medical Oncology, Department of Medicine, Surgery and Neurosciences, University of Siena, Siena, 53100 Italy; 6 Department of Medicine, Surgery and Health Sciences, University of Trieste, Trieste, Italy; 7 Multidisciplinary Unit of Breast Pathology and Translational Research, Cremona Hospital, Cremona, Italy

**Keywords:** Cyclin-dependent kinase 4/6, Advanced breast cancer, Hormone positive breast cancer, Adverse events, Efficacy outcomes

## Abstract

**Background:**

CDK4/6 inhibitors have transformed treatment for HR + HER2 − advanced breast cancer (aBC). However, adverse events (AEs) often lead to dose adjustments or discontinuation, potentially impacting outcomes. This study assessed AE incidence and its effect on survival in patients receiving abemaciclib (AB), ribociclib (RB), or palbociclib (PB).

**Methods:**

A retrospective study of 162 h + HER2 − aBC patients treated with CDK4/6 inhibitors as first-line therapy (July 2017–September 2024) was conducted. AE incidence, progression-free survival (PFS), and overall survival (OS) were analyzed.

**Results:**

Most patients (91.4%) were postmenopausal, with a median follow-up of 24.6 months. AEs occurred in 87% of patients, with grade 3–4 neutropenia most common in PB (79.3%) and RB (80%), while AB caused more diarrhea (66.7%). Dose reductions due to AEs were linked to significantly longer PFS (38.5 vs. 16.3 months, *p* < 0.001) and OS (NR vs. 32.4 months, *p* = 0.024). Treatment discontinuation was highest for PB (19.6%), followed by RB (16.9%) and AB (14.9%).

**Conclusions:**

CDK4/6 inhibitors have distinct toxicity profiles. Effective AE management and dose adjustments are crucial for maintaining efficacy, emphasizing the need for AE prediction models to optimize CDK4/6i use in HR + HER2 − aBC.

**Supplementary Information:**

The online version contains supplementary material available at 10.1007/s00280-025-04836-y.

## Introduction

Breast cancer (BC) is the most prevalent and fatal cancer type among women globally [1]. However, over recent decades, significant advancements in scientific research and pharmacological treatments have markedly improved median survival rates for affected patients. The treatment of advanced BC (aBC) currently relies on systemic therapies, selected based on the tumor’s biological subtype [2]. Approximately 75% of cases are hormone receptor-positive (HR+) and human epidermal growth factor receptor 2-negative (HER2−) [3–5].

The introduction of cyclin-dependent kinase 4/6 (CDK4/6) inhibitors has transformed the management of HR + BC, with robust evidence supporting their efficacy when combined with endocrine therapy [6]. The CDK-RB1-E2F pathway, a key regulator of cell cycle progression, is commonly disrupted in cancer. In BC, proliferative signals, including estrogen receptor activation, stimulate the association of CDK4/6 with cyclin D1. Combining CDK4/6 inhibitors with endocrine therapies enhances therapeutic efficacy by downregulating cyclin D1 expression, thereby reducing CDK4/6 complex formation and inhibiting tumor growth (ref).

Currently, CDK4/6 inhibitors such as abemaciclib (AB), ribociclib (RB), and palbociclib (PB) are established as standard treatments. They are employed as first-line therapies in combination with aromatase inhibitors or as second-line therapies in combination with fulvestrant [5, 7–11]. Several observational studies in real-world settings have corroborated the positive impact of CDK4/6 inhibitors on patient outcomes [12, 13]. To date, however, no direct comparative studies have been conducted among these three CDK4/6 inhibitors for HR+, HER2 − advanced breast cancer. Presently, the choice of a specific CDK4/6 inhibitor is informed by clinical experience, variations in toxicity profiles, cost considerations, and institutional prescribing policies.

Despite the effectiveness of CDK4/6 inhibitors, a significant proportion of patients undergoing treatment experience adverse events (AEs). These AEs often require dose adjustments or, in more severe cases, may lead to treatment discontinuation. Such events can impair organ or tissue function and, in some instances, may present life-threatening risks, highlighting the critical need for continuous monitoring throughout the therapy.

In this study, we performed a real word data based retrospective analysis to investigate the incidence of AEs in patients with mBC treated with CDK4/6 inhibitors as AB, RB, and PB and if they are aligned with those reported in RCT. Moreover, we also evaluated the prognostic impact of the AEs on PFS and OS to provide new potential insights for the optimization of the CDK4/6Is use in HR+/HER2- patient management.

## Materials and methods

### Patient selection and study design

Patients with HR + HER2- mBC who had received at least one administration of a CDK4/6 inhibitor (AB, RB, or PB) as first-line therapy between January 2019 and September 2024 in different oncology centers were included in the study. Inclusion criteria required the availability of demographic and clinical parameters, including menopausal status, receptor status, Eastern Cooperative Oncology Group performance status (ECOG PS), metastatic sites, comorbidities, and type of therapy. The unavailability of such data constituted an exclusion criterion.

Patients were classified into three groups based on the specific treatment regimen: AB, RB, and PB.

The study protocol was approved by the Ethics Committee of ATS Val Padana (Eudract 2018–002116); written informed consent was obtained from all patients included in the analysis. The research adhered to the principles set forth in the Declaration of Helsinki of 1964 and its subsequent amendments. Clinical and laboratory data were sourced from electronic databases and medical records.

### Treatment protocol and assessment

Patients received specific treatment regimens in accordance with the European Medicines Agency’s product information. Dose adjustments, treatment delays, and interruptions were determined by the investigator based on clinical judgment and standard practice.

Follow-up typically included computed tomography scans of the chest and abdomen, and bone scintigraphy as needed, at 12-week intervals. During treatment, monitoring included blood tests and clinical visits every 4 weeks. Treatment continued until radiographic or clinical disease progression or the onset of intolerable AEs. Tumor response was assessed using RECIST criteria version 1.1 [14].

The severity of AEs was evaluated according to the Common Terminology Criteria for Adverse Events (CTCAE) version 5 [15]. Specific AEs included hematologic, gastrointestinal, hepatobiliary, and cardiologic events, as well as rarer occurrences such as deep vein thrombosis, skin rash, and mucositis.

### Efficacy outcomes and statistical analysis

Progression-free survival (PFS) was defined as the duration from treatment initiation to disease progression or death, while overall survival (OS) was defined as the interval between treatment initiation and death from any cause. PFS and OS were compared between patients who developed AEs of any grade or high-grade AEs (G3-4) and those who did not or low-grade (G1-2), as well as between patients who required treatment discontinuation or dose reduction due to AEs and those who did not. A landmark analysis was performed at 3 and 6 months from treatment initiation to evaluate the impact of early dose reduction on PFS and OS, including only patients who were alive and progression-free at each respective landmark time point.

Continuous variables were analyzed using the Mann-Whitney U test, and categorical variables were evaluated using Fisher’s exact test. Patients lost to follow-up were censored at their last known contact. Survival data up to the end of September 2024 were analyzed; PFS and OS were estimated using the Kaplan-Meier method and compared using the log-rank test. Hazard ratios (HRs) with 95% confidence intervals (CIs) and statistical significance (*p* ≤ 0.05) were incorporated into the statistical analysis plan.

## Results

### Patient characteristics

Among patients with HR + HER2- mBC treated with CDK4/6 inhibitors as first-line therapy, 162 patients were deemed eligible and included in the study, all of whom were female. Most of the patients were postmenopausal (91.4%), while a smaller proportion were premenopausal (8.6%). All patients included in the study had tumors expressing estrogen receptor (ER+), while progesterone receptor expression was observed in 87.6% of cases. Additionally, most patients were HER2- (78.4%), with a minority presenting non-amplified HER2 (18.5%) or HER2-positive status (3.1%).

At the initiation of CDK4/6 inhibitor treatment, 35 patients (42.0%) exhibited elevated carcinoembryonic antigen (CEA), and 58 (69.7%) showed increased cancer antigen 15 − 3 (Ca-15.3) levels. More than half of the patients (66.0%) had an ECOG-PS of 0. Bone metastases were identified in 47 patients (56.2%), while pulmonary and lymph node involvement were observed in 36.4% and 50.0% of patients, respectively. Liver metastases were present in 26.50% of cases, and brain metastases were noted in 3.7%.

A total of 125 patients (77.2%) had undergone prior surgical procedures, with an equal percentage also receiving radiotherapy and hormone therapy. Regarding first-line CDK4/6 inhibitor therapy, 47 patients (29%) received AB, 56 (34.6%) received RB, and 59 (36.4%) were treated with PB. Endocrine therapy combined with CDK4/6 inhibitors was letrozole in 80 patients (49.4%), anastrozole in 6 (3.7%), exemestane in 5 (3.08%), and fulvestrant in 71 (43.82%) patients. In 13 women, a luteinizing hormone-releasing hormone receptor (LHRH) analog was also added. Finally, 77 patients (47.5%) received bone-targeting therapy with bisphosphonates or denosumab.

The baseline characteristics of the patients are summarized in Table 1.

**Table 1:** Patient’s characteristics

Variables	Patients, *n* (%) (*N* = 162)
Sex, n (%) Female	162 (100)
ECOG, n (%) 0 ≥ 1	107 (66.0) 55 (34.0)
Menopausal status, n (%) Pre-menopausal Post-menopausal	14 (8.6) 148 (91.4)
ER+, n (%) Yes	162 (100)
PgR+, n (%) Yes	142 (87.6)
HER2, n (%) Negative Not amplified Positive	127 (78.4) 30 (18.5) 5 (3.1)
Surgery, n (%) Yes	125 (77.2)
Radiotherapy, n (%) Yes	127 (78.4)
Previous hormone therapy, n (%) Yes	123 (75.9)
Hormone therapy associated, n (%) Letrozole Anastrazole Exemestan Fulvestrant +- LHRHa	80 (49.4) 6 (3.70) 5 (3.08) 71 (43.82) 13 (8.02)
CEA, n (%) Out of range	68 (42.0)
Ca-15.3, n (%) Out of range	113 (69.7)
Metastasis, n (%) Bone Lung Lymphonodes Liver Brain	91 (56.2) 59 (36.4) 81 (50.0) 43 (26.5) 6 (3.7)
Type CDK4/6, n (%) Abemaciclib Ribociclib Palbocicib	47 (29.0) 56 (34.6) 59 (36.4)
Bisphosphonates Yes	77 (47.5)

ECOG, Eastern cooperative oncology Group; ER, Estrogen receptor; PgR, progesterone receptor; HER2, human epidermal growth factor receptor 2; CEA, Carcino-Embryonic Antige; Ca, cancer antigen; CDK, cyclin-dependent kinase

### Adverse events

Among the three groups, 41 out of 47 patients (87.2%) in the AB group, 51 out of 56 (91.1%) in the RB group, and 49 out of 59 (83.0%) in the PB group experienced at least one adverse event (AE) of any grade.

Regarding specific AEs, significant differences were observed for diarrhea of any grade across the three groups (AB: 66.0%, RB: 3.4%, PB: 8.9%, *p* < 0.001), for neutropenia of any grade (AB: 40.4%, RB: 79.7%, PB: 80.4%, *p* < 0.001), thrombocytopenia of any grade (AB: 29.8%, RB: 13.6%, PB: 32.1%, *p* = 0.04) and increasing AST/ALT of any grade (AB: 36.2%, RB: 25.4%, PB: 10.7%, *p* = 0.008) as well as grade 3–4 neutropenia (AB: 25.5%, RB: 35.6%, PB: 62.5%, *p* < 0.001), grade 3–4 anemia (AB: 8.5%, RB: 0%, PB: 0%, *p* < 0.001), and grade 3–4 diarrea (AB: 10.6%, RB: 3.4%, PB: 0%, *p* = 0.03). Detailed data regarding AEs are collected in Table 2.

**Table 2:** Adverse events in immune combinations therapy groups

	Abemaciclib, *n* (%)	Ribociclib, *n* (%)	Palbociclib, *n* (%)	*P*
Any	41 (87.2)	51 (91.1)	49 (83.0)	0.440
Anemia Any G3-4	15 (32.0) 4 (8.5)	14 (23.7) 0	11 (19.6) 0	0.347 **< 0.001**
Neutropenia Any G3-4	19 (40.4) 12 (25.5)	47 (79.7) 21 (35.6)	45 (80.4) 35 (62.5)	**< 0.001** **< 0.001**
Thrombocytopenia Any G3-4	14 (29.8) 0	8 (13.6) 2 (3.4)	18 (32.1) 2 (3.6)	**0.04** 0.55
Diarreha Any G3-4	31 (66.0) 5 (10.6)	2 (3.4) 2 (3.4)	5 (8.9) 0	**< 0.001** **0.03**
AST/ALT increase Any G3-4	17 (36.2) 2 (4.3)	15 (25.4) 0	6 (10.7) 2 (3.6)	**0.008** 0.325
CardioTox Yes	8 (17.0)	14 (23.7)	5 (8.9)	0.100
QT lengthening Yes	4 (8.5)	4 (6.8)	0	0.074

AST, Aspartate Transferase; ALT, Alanine Transaminase

Treatment discontinuation occurred in 7 patients (14.9%) in the AB group, with 19 patients (40.4%) requiring dose reductions. In the RB group, 10 patients (16.9%) discontinued treatment, and 20 (33.9%) reduced the dose. In the PB group, 11 patients (19.6%) discontinued, and 26 (46.4%) underwent dose reduction. Reductions did not reach statistical significance between the three groups, as well as discontinuation (*p* = 0.39 and *p* = 0.81, respectively). The incidence of treatment interruptions and reductions is detailed below in table S1.

### Survival outcomes

The association between the development of AEs and PFS and OS after the initiation of treatment was analyzed.

PFS was comparable between patients with AEs of any grade and those without (median: 24.6 months, 95% CI: 19.6–32.8 vs. 56.4 months, 95% CI: 2.7–NR; *p* = 0.95). Likewise, OS was similar in patients with AEs of any grade compared to those without (median: 41.0 months, 95% CI: 32.0–46.8 vs. NR, 95% CI: 13.4–NR; *p* = 0.51) (Figure S1).

Among patients who developed AEs, PFS and OS was comparable between those with grade 3–4 AEs and those with grade 1–2 AEs (PFS: median: 29.1 months, 95% CI: 19.7–35.8 vs. 19.7 months, 95% CI: 16.2–39.8; *p* = 0.18; OS: median: 41.0 months, 95% CI: 31.2–NR vs. 42.4 months, 95% CI: 32.4–NR; *p* = 0.72) (Figure S1).

**Fig. 1:**
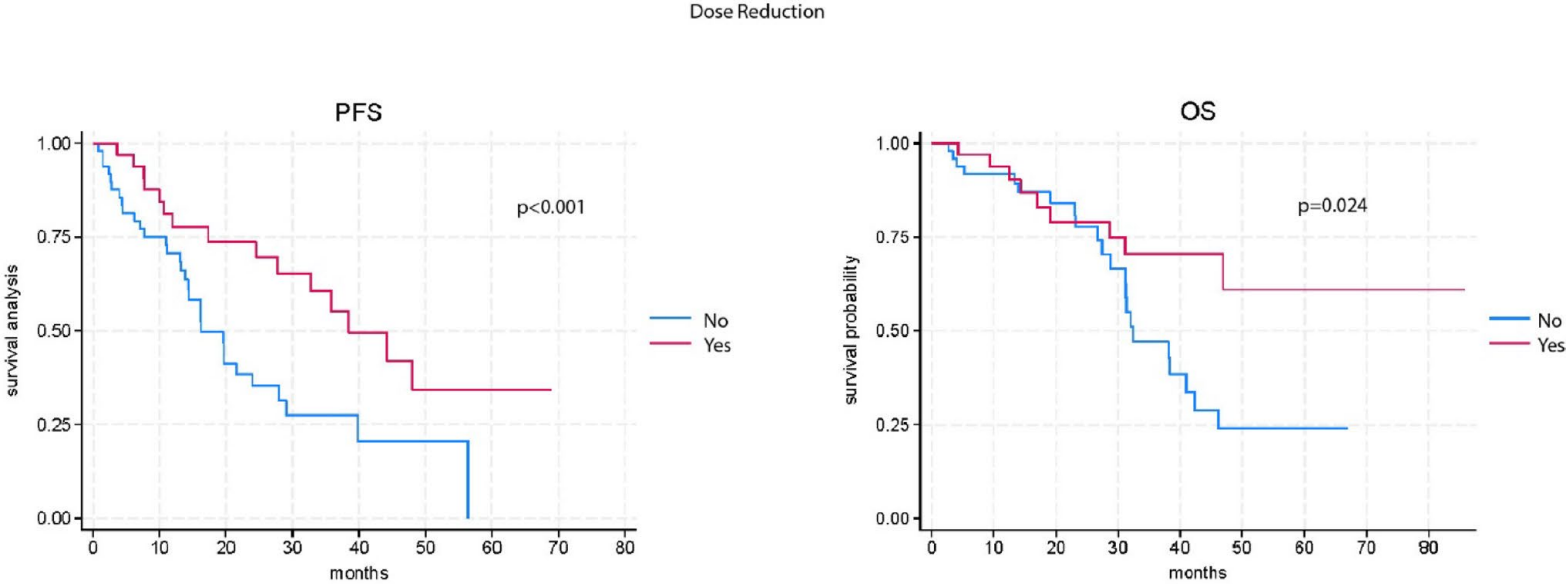
Kaplan-Meier survival curve of progression free survival (PFS) and overall survival (OS) according to therapy dose reduction

A statistically significant difference in PFS and OS was observed between patients who had a dose reduction of CDK4/6 inhibitor due to adverse events (AEs) and those who did not. Specifically, the median PFS was 38.5 months (95% CI: 32.8-NR) in patients who experience dose reduction, compared to 16.3 months (95% CI: 14.4–21.5) in those who did not (*p* < 0.001). Similarly, the median OS was not reached (NR) in the group with dose reduction (95% CI: 46.8-NR) versus 32.4 months (95% CI: 31.1–41.0) in the group without dose-reduced (*p* = 0.024) (Fig. 1).

**Fig. 2:**
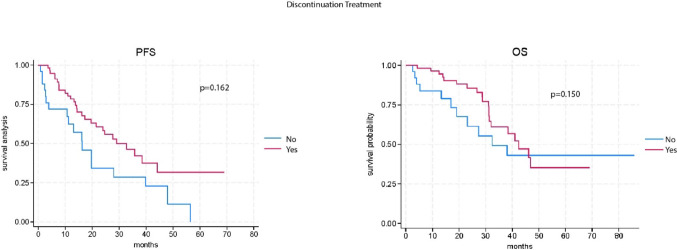
Kaplan-Meier survival curve of progression free survival (PFS) and overall survival (OS) according to treatment discontinuation

However, PFS and OS were similar between patients who discontinued therapy and those who did not in another subgroup. Specifically, the median PFS was 32.8 months (95% CI: 26.4–48.0) in patients who discontinued therapy versus 21.5 months (95% CI: 16.3–29.1) in those who did not (*p* = 0.16). The median OS was not reached (NR) for patients who discontinued therapy (95% CI: 31.1-NR), compared to 38.3 months (95% CI: 31.4–46.2) for those who did not discontinue (*p* = 0.15) (Fig. 2).

Data regarding PFS and OS are provided in Table 3.

**Table 3:** Univariate and multivariate analysis for PFS

	HR	IC 95%	*P*
**Univariate**			
ECOG ≥ 1 (yes vs. no)	**1.68**	**1.11–1.53**	**0.013**
Menopausal status (Post-menopausal vs. pre-menopausal)	0.94	0.55–1.89	0.832
HER2 positive (yes vs. no)	1.45	0.86–2.15	0.783
Surgery (yes vs. no)	1.03	0.67–1.95	0.876
Radiotherapy (yes vs. no)	1.25	0.76–3.11	0.678
Previous hormone therapy (yes vs. no)	1.55	0.65–2.99	0.789
Hormone therapy associated (fulvestrant vs. other)	0.87	0.66–2.34	0.654
CEA out of range (yes vs. no)	1.89	0.77–2.25	0.587
Ca-15.3 out of range (yes vs. no)	1.35	0.89–1.87	0.112
Bone metastases (yes vs. no)	**2.24**	**1.40–3.59**	**0.001**
Lung metastasis (yes vs. no)	0.72	0.47–1.12	0.156
Lymphonodes metastases (yes vs. no)	0.82	0.54–1.24	0.353
Liver metastases (yes vs. no)	0.94	0.58–1.93	0.828
Brain metastases (yes vs. no)	**8.41**	**3.48–20.3**	**< 0.001**
Bisphosphonates (yes vs. no)	1.47	0.96–2.24	0.072
Dose reduction (yes vs. no)	**0.43**	**0.27–0.68**	**< 0.001**
**Multivariate**			
ECOG ≥ 1 (yes vs. no)	1.35	0.88–2.08	0.161
Bone metastases (yes vs. no)	**1.84**	**1.13–3.00.13.00**	**0.014**
Brain metastases (yes vs. no)	**4.96**	**2.02–12.01**	**< 0.001**
Dose reduction (yes vs. no)	**0.49**	**0.31–0.78**	**0.003**

ECOG, Eastern Cooperative Oncology Group; HER2, human epidermal growth factor receptor 2; CEA, Carcino-Embryonic Antige; Ca, cancer antigen; CDK, cyclin-dependent kinase

In the univariate Cox regression analysis for PFS, several clinical variables showed a significant association with patient outcomes. ECOG PS ≥ 1 (HR 1.68, 95% CI 1.11–1.53, *p* = 0.013), the presence of bone metastases (HR 2.24, 95% CI 1.40–3.59, *p* = 0.001), and brain metastases (HR 8.41, 95% CI 3.48–20.3, *p* < 0.001) were associated with a higher risk of progression, while dose reduction was associated with a significantly lower risk (HR 0.43, 95% CI 0.27–0.68, *p* < 0.001). In the multivariate model, bone metastases (HR 1.84, 95% CI 1.13–3.00, *p* = 0.014), brain metastases (HR 4.96, 95% CI 2.02–12.01, *p* < 0.001), and dose reduction (HR 0.49, 95% CI 0.31–0.78, *p* = 0.003) remained independently associated with PFS (Table 4).

**Table 4:** Univariate and multivariate analysis for OS

	HR	IC 95%	*P*
**Univariate**			
ECOG ≥ 1 (yes vs. no)	**2.24**	**1.36–3.68**	**0.002**
Menopausal status (Post-menopausal vs. pre-menopausal)	0.77	0.45–1.58	0.763
HER2 positive (yes vs. no)	1.89	0.78–2.15	0.875
Surgery (yes vs. no)	0.99	0.78–1.56	0.678
Radiotherapy (yes vs. no)	1.03	0.78–2.11	0.786
Previous hormone therapy (yes vs. no)	1.35	0.934–3.11	0.892
Hormone therapy associated (fulvestrant vs. other)	0.55	0.33–1.98	0.756
CEA out of range (yes vs. no)	1.45	0.92–2.76	0.565
Ca-15.3 out of range (yes vs. no)	1.25	0.87–4.54	0.634
Bone metastases (yes vs. no)	**3.22**	**1.71–6.05**	**< 0.001**
Lung metastasis (yes vs. no)	0.73	0.43–1.23	0.247
Lymphonodes metastases (yes vs. no)	1.21	0.73–2.00.73.00	0.444
Liver metastases (yes vs. no)	0.96	0.54–1.72	0.915
Brain metastases (yes vs. no)	**5.94**	**2.51–14.09**	**< 0.001**
Bisphosphonates (yes vs. no)	**1.67**	**0.99–2.81**	**0.053**
Dose reduction (yes vs. no)	**0.45**	**0.26–0.79**	**0.006**
**Multivariate**			
ECOG ≥ 1 (yes vs. no)	1.56	0.91–2.67	0.100
Bone metastases (yes vs. no)	2.29	0.96–5.47	0.062
Brain metastases (yes vs. no)	**3.29**	**1.11–9.70**	**0.030**
Bisphosphonates (yes vs. no)	1.12	0.51–2.43	0.769
Dose reduction (yes vs. no)	**0.57**	**0.32–0.99**	**0.050**

ECOG, Eastern Cooperative Oncology Group; HER2, human epidermal growth factor receptor 2; CEA, Carcino-Embryonic Antige; Ca, cancer antigen; CDK, cyclin-dependent kinase

Similar patterns were observed for OS: in the univariate analysis, ECOG PS ≥ 1 (HR 2.24, 95% CI 1.36–3.68, *p* = 0.002), bone metastases (HR 3.22, 95% CI 1.71–6.05, *p* < 0.001), brain metastases (HR 5.94, 95% CI 2.51–14.09, *p* < 0.001), and dose reduction (HR 0.45, 95% CI 0.26–0.79, *p* = 0.006) were significantly associated with OS. In the multivariate model, brain metastases (HR 3.29, 95% CI 1.11–9.70, *p* = 0.030) and dose reduction (HR 0.57, 95% CI 0.32–0.99, *p* = 0.050) remained significant (Table 5).

**Table 5:** PFS and OS according to adverse events

	Median PFS, months (95% CI)	*P*	Median OS, months (95% CI)	*P*
All patients (*N* = 162)	24.6 (19.6–32.8)		40.9 (32.4-NR)	
Any grade AEs Yes (141) No (21)	24.6 (19.6–32.8) 56.4 (2.7-NR)	0.950	40.6 (32.0–46.8.0.8) NR (13.4-NR)	0.511
Grade 3–4 AEs No (81) Yes (81)	19.7 (16.2–39.8) 29.1 (19.7–35.8)	0.181	42.4 (32.4-NR) 41.0 (31.2-NR)	0.723
Dose reduction Yes (65) No (97)	38.5 (32.8-NR) 16.3 (14.4–21.5)	**< 0.001**	NR (46.8-NR) 32.4 (31.2–41.0)	**0.024**
Treatment discontinuation Yes (28) No (134)	32.8 (24.6–48) 21.5 (16.3–29.1)	0.162	NR (31.1-NR) 38.3 (31.4–46.2)	0.150

PFS, progression free survival; OS, overall survival; AEs, Adverse events; CI, confidence interval

**Fig. 3:**
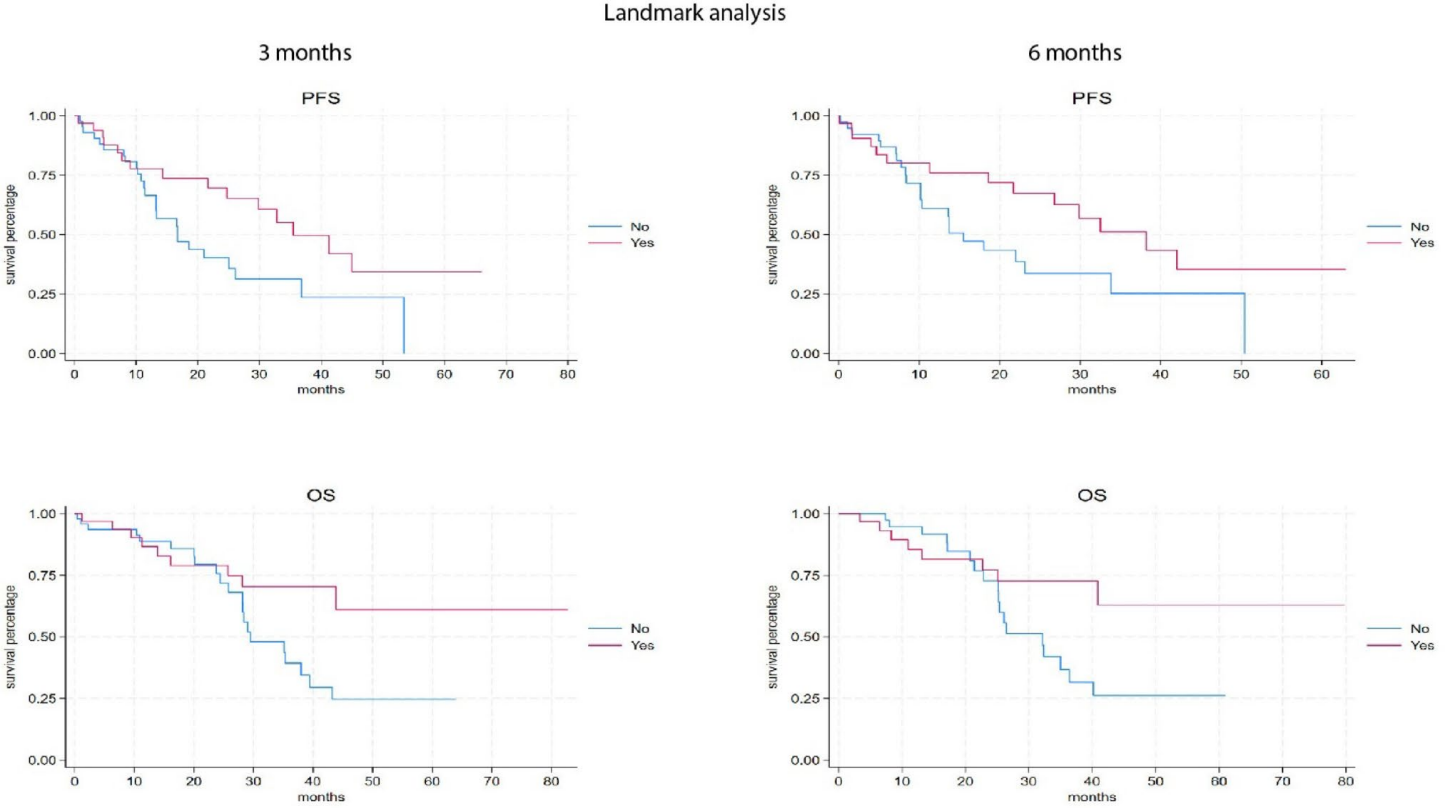
Kaplan-Meier survival curve of progression free survival (PFS) and overall survival (OS) according to 3 and 6 months Landmark analysis

Landmark analyses further supported these findings, showing that patients who underwent dose reduction had significantly longer median PFS and OS at both 3 and 6 months compared with those without dose reduction (Table S2and Fig. 3).

## Discussion

The incidence of AEs in patients with HR + HER2 − aBC treated with first-line CDK4/6 inhibitors remains a clinically relevant challenge. Over 60% of patients required at least one treatment interruption or delay, and more than 30% needed a dose reduction [16]. This aligns with phase 3 studies, where 40–50% of patients required dose adjustments within the first or second treatment cycle [7–11]. In our cohort, 87% of patients developed at least one AE of any grade with permanent discontinuation more frequently among patients treated with PB (19.6%) compared to RB (16.9%) and AB (14.9%). These discontinuations were primarily attributed to hematologic toxicity for PB and RB, and gastrointestinal AEs for AB.

Our findings align with the safety profiles reported in RCT data, confirming high rates of hematologic AEs with PB and RB and gastrointestinal AEs with AB. However, notable differences exist between the three agents.

A recent large-scale analysis examined the AEs associated with CDK4/6 inhibitors reported in the Food and Drug Administration Adverse Event Reporting System from 2015 to 2022 [17]. The adverse reactions most reported for PB are neutropenia, infections, leukopenia, fatigue, and nausea. RB is frequently associated with AEs such as myelosuppression and liver function abnormalities, including laboratory findings. Likewise, AB is primarily linked to adverse reactions such as diarrhea, neutropenia, nausea, abdominal pain, and infections [17]. In our analysis, grade 3–4 neutropenia was the most common AE with PB (79.3%) and RB (80%), whereas it was less frequent with AB (41.7%).

Neutropenia typically occurred early, within the first treatment cycle, with a median onset at 28 days and a duration of approximately 7 days. Grade 3 diarrhea was more frequent with AB (66.7%) compared to PB (10.3%) and RB (3.3%), peaking in the first cycle and decreasing thereafter. Managing diarrhea is critical due to its potential impact on drug absorption. A pharmacokinetic model suggested that AB doses of 200 mg twice daily led to reduced drug absorption due to increased gastrointestinal toxicity, with mitigation strategies including dose adjustments and supportive care. RB is uniquely associated with QTc prolongation, with a higher incidence observed in combination with tamoxifen (16%) versus aromatase inhibitors (7%) in the MONALEESA-7 trial [18]. QTc monitoring is recommended, especially when co-administering drugs with QT-prolonging potential.

A recent systematic review and meta-analysis evaluated dose reduction and discontinuation rates associated with CDK4/6 inhibitors in combination with endocrine therapy across several clinical settings [19]. Including data from 15 randomized and real-world studies, the analysis found no compromise in efficacy, as measured by PFS and OS, despite dose adjustments due to AEs. Notably, PB and RB exhibited similar dose-reduction profiles, while AB showed marginally higher discontinuation rates, primarily due to gastrointestinal toxicities. The meta-analysis reinforces the concept that treatment management, rather than discontinuation, is key to maintaining therapeutic benefits in patients experiencing toxicity [19]. Other two recent studies reported the absence of disparity in PFS between patients with dose reduction and those without, due to AEs [20, 21]. These findings support clinical practice strategies that prioritize proactive dose modifications and toxicity monitoring, aligning with our real-world observations and emphasizing the feasibility of individualized CDK4/6 inhibitor regimens. An intriguing and unexpected finding of our analysis was the observation that patients who required dose reductions of CDK4/6 inhibitors experienced significantly improved survival outcomes compared with those who maintained the standard dosing schedule. Several hypotheses may account for this result. One possibility is that patients developing early adverse events may have higher systemic drug exposure, reflecting increased pharmacodynamic sensitivity to CDK4/6 inhibition, which in turn could translate into enhanced antitumor efficacy. Alternatively, dose adjustments may have facilitated better long-term treatment adherence by improving tolerability and reducing the risk of permanent discontinuation, ultimately extending progression-free and overall survival. Another explanation might be related to underlying biological or clinical characteristics predisposing certain patients both to toxicity and to more favorable therapeutic outcomes. Nonetheless, given the limited sample size and retrospective design, these findings should be considered hypothesis-generating and interpreted with caution until validated in larger, prospective studies. Our findings appear to contrast with prior reports suggesting that early dose reductions of CDK4/6 inhibitors are associated with poorer clinical outcomes, including shorter PFS and OS [22–24]. Several factors may explain this apparent discrepancy. First, the timing of dose modification may differ across studies: whereas early reductions in randomized controlled trial settings might reflect inadequate drug exposure, in our real-world cohort dose adjustments may have occurred later and allowed patients to remain on treatment for a longer duration. Second, heterogeneity in patient populations and study design, particularly between prospective trials and retrospective observational cohorts, may contribute to divergent results. Finally, residual confounding cannot be excluded, as patients who tolerated treatment sufficiently to warrant dose reductions rather than discontinuation may inherently have had a more favorable prognosis. Taken together, these differences highlight the need for further investigation to clarify the complex relationship between dose intensity, toxicity management, and long-term outcomes in patients treated with CDK4/6 inhibitors.

A further consideration is that the apparent survival advantage associated with dose reductions in our cohort may be influenced by immortal time bias. Because dose reduction is a time-dependent event, patients must survive long enough to experience it; consequently, failure to account for this factor could artificially inflate survival estimates. Ideally, this issue should be addressed through time-dependent covariate analysis in Cox regression models, which was not feasible within the scope of the present study. Therefore, our findings regarding the prognostic impact of dose reduction should be interpreted with caution and regarded as exploratory.

Another important point is the observed median PFS of 38.5 months in the dose-reduced group, which is markedly higher than the 24–28 months typically reported in pivotal phase III trials [7–11]. Several factors may explain this discrepancy. Our study population may have been subject to selection bias, with patients presenting more favorable baseline characteristics or lower disease burden. Differences in prior treatments and endocrine sensitivity status could also have contributed. Additionally, as our analysis was conducted in a real-world setting with limited follow-up assessments compared to randomized controlled trials, methodological factors may have led to an overestimation of PFS. These aspects highlight the importance of cautious interpretation and the need for further validation in larger, prospective datasets.

This study has several limitations that warrant consideration. First, the relatively small sample size that limited the statistical power of subgroup analyses and the retrospective design that inherently introduces the possibility of selection bias and incomplete data capture, which could affect the accuracy of adverse event reporting and survival outcomes. Second, although we attempted to account for relevant clinical characteristics, we were unable to fully adjust for potential confounders such as comorbidities, concomitant medications, or differences in prior treatments, all of which might have influenced both toxicity profiles and prognosis. Third, the timing and criteria for dose reductions or discontinuations were based on physician judgment in routine practice and were not standardized, potentially introducing heterogeneity into the interpretation of survival outcomes. Finally, another limitation is the potential for immortal time bias in the analysis of dose reduction and survival outcomes. Since dose reduction is a time-dependent event, patients must survive long enough to experience it, which could artificially inflate survival estimates. To reduce this bias, we performed Cox regression including dose reduction as a time-dependent covariate and conducted landmark analyses at 3 and 6 months, both of which confirmed the robustness of our findings. Taken together, these limitations underscore the need for validation of our results in larger, prospective, and methodologically uniform cohorts.

## Conclusion

This analysis provides valuable insights into the incidence, spectrum, and prognostic significance of AEs associated with first-line treatment of HR + HER2 − aBC using CDK4/6 inhibitors. The findings corroborate evidence from clinical trials, highlighting the high prevalence of AEs and the dose reductions, rather than discontinuation, may confer survival benefits. Importantly, this study also underscores key differences in the toxicity profiles of these inhibitors, which can guide clinical decision-making. These findings reinforce the importance of personalized AE management strategies to optimize CDK4/6 inhibitor use in HR + HER2 − aBC.

## Supplementary Information

Below is the link to the electronic supplementary material.

Supplementary Material 1

Supplementary Material 2: Kaplan-Meier survival curve of progression free survival (PFS) and overall survival (OS) according to adverse events (AEs)

Supplementary Material 3

## Data Availability

No datasets were generated or analysed during the current study.
