# Supplementary material for: Impact of adverse events on survival outcomes in patients treated with CDK4/6 inhibitors for advanced breast cancer
Source: Cancer Chemother Pharmacol. 2025 Dec 3;95(1):117. doi: 10.1007/s00280-025-04836-y (PMC12672692; doi:10.1007/s00280-025-04836-y)
Supplement: Supplementary file 1 — Supplementary Material 1 [file 280_2025_4836_MOESM1_ESM.docx]

**Table S1**. CDK 4/6 dose reduction or discontinuation.

|  | Abemaciclib | | Ribociclib | | Palbociclib | | P |
| --- | --- | --- | --- | --- | --- | --- | --- |
|  | Yes | No | Yes | No | Yes | No |  |
| Reduction  Our study  RCT | 19 (40.4)  142 (43.4)^1^ | 28 (59.6) | 20 (33.9)  NA^2^ | 39 (66.1) | 26 (46.4)  160 (36.0)^3^ | 30 (53.6) | 0.393 |
| Discontinuation  Our study  RCT | 7 (14.9)  64 (19.6)^1^ | 40 (85.1) | 10 (16.9)  37 (11.1)^2^ | 49 (83.1) | 11 (19.6)  33 (7.5)^3^ | 45 (80.4) | **0**.813 |

*CDK, cyclin-dependent kinase; RCT, randomized controlled trial. Refered to MONARCH 3 trial^1^, MONALEESA 2^2^, PALOMA 2^3^*
