# Supplementary material for: Impact of adverse events on survival outcomes in patients treated with CDK4/6 inhibitors for advanced breast cancer
Source: Cancer Chemother Pharmacol. 2025 Dec 3;95(1):117. doi: 10.1007/s00280-025-04836-y (PMC12672692; doi:10.1007/s00280-025-04836-y)
Supplement: Supplementary file 3 — Supplementary Material 3 [file 280_2025_4836_MOESM3_ESM.docx]

**Table S2:** Landmark analysis at three and 6 months for PFS and OS.

|  | **Median PFS, months (95% CI)** | **P** | **Median OS, months (95% CI)** | **P** |
| --- | --- | --- | --- | --- |
| **Landmark analysis 3 months** | | | | |
| **Dose reduction**  Yes  No | 35.5 (13.2-25.0)  16.7 (13.2-NR) | 0.01 | NR (43.8-NR)  29.4 (28.2-37.9) | 0.004 |
| **Landmark abalysis 6 months** | | | | |
| **Dose reduction**  Yes  No | 38.2 (26.8-NR)  15.5 (10.3-23.1) | 0.02 | NR (40.8-NR)  32.1 (25.2-36.4) | 0.07 |

*PFS, progression free survival; OS, overall survival; CI, confidence interval; NR, not reached.*
